# The Relationship between Self-Employed Workers’ Entrepreneurial Attitude and Health Status

**DOI:** 10.3390/ijerph17061892

**Published:** 2020-03-14

**Authors:** José Antonio Climent-Rodríguez, Yolanda Navarro-Abal, Celia Sánchez-López, Agustín Galán-García, Juan Gómez-Salgado

**Affiliations:** 1 Faculty of Health Sciences, University Isabel I, 09003 Burgos, Spain; jose.climent@dpsi.uhu.es; 2 School of Labour Sciences, University of Huelva, 21007 Huelva, Spain; celia@ole.uhu.es (C.S.-L.); agustin@uhu.es (A.G.-G.); 3 HISLABINST Investigation Group, University of Seville, 41004 Sevilla, Spain; 4 School of Labour Sciences, Department of Sociology, Social Work and Public Health, University of Huelva, 21007 Huelva, Spain; salgado@uhu.es; 5 Safety and Health Posgrade Program, University Espiritu Santo, Guayaquil 092301, Ecuador

**Keywords:** occupational health promotion, innovative and intrapreneurial skills, quality of working life, engaged workers, work-health balance, mental health, occupational health

## Abstract

In the new work environment, self-employment as a formula and entrepreneurship as an attitude have gained prominence as a means to foster a more competitive economy and increase employment opportunities. Having an entrepreneurial attitude, in addition to being positive on a work level, can also have positive effects on the health of the entrepreneur. The objective of this study is to analyze the relationship between an entrepreneurial attitude and its influence on the general health of Spanish self-employed workers who possess these skills, compared to those who lack them. A cross-sectional descriptive study was carried out through random sampling of 1148 self-employed professionals throughout Spain from 21 different economic sectors. The results show the existence of a negative relationship between entrepreneurial attitude and age, that is to say, an entrepreneurial attitude decreases with age. With the sex variable, women show less entrepreneurial attitude and with mental health, decreased mental health was shown in those with a greater entrepreneurial attitude. On the other hand, there is a positive linearity between a positive attitude and the social function of the state of health.

## 1. Introduction

The new work environment is encouraging the development of alternative employment models due to the crisis in the salaried employment system. It is increasingly common for people to face difficulties working for someone else, and this is giving rise to a new generation of workers who, in order to be employed, create their own employment opportunity. The concept of entrepreneurship has gained prominence in recent years as a means to foster a more competitive economy and increase the chances of employability [1].

Although the self-employed person has occupied an important place in the employment scene for decades, this figure has been poorly valued socially and administratively, probably due to the predominance of cultural and socio-labour models based on working for someone else and public employment. However, in recent years, the concept has gradually changed towards approaches that focus on self-employment as a formula and entrepreneurship as an attitude.

There are many different definitions related to the concept of entrepreneurship. The entrepreneur has been described [2] from different perspectives by historians, economists, and sociologists, but even so, there does not seem to be an agreement on the variables that define it. Some definitions attribute the meaning of “being in charge” or “undertaking something” to it, others refer to “the ability to create and build something from nothing,” or relate it to those people who are able to feel that creating something new is an opportunity and not a problem.

Over time, the tendency to equate the concept of an entrepreneurial person with a business-person has been consolidated. Thus, Concha and Muñoz [3] describe the entrepreneur, adapting it to the notion of company and businessperson and estimating, in broad strokes, that the action of creating a company involves setting objectives and the commitment of certain resources for its achievement. On the other hand, some studies [4,5] use the term businessperson and entrepreneur interchangeably. Other authors, such as Prat [6], believe that the entrepreneur, unlike the business-person, has a more active attitude and is more daring, bold, determined, and decisive.

Entrepreneurship is not always synonymous with self-employment. Some studies [7,8] also reveal how entrepreneurship is an internal component that is related to the personality traits of each individual, rather than solely an external behavioural process like setting up a company. This is why we have started talking about an entrepreneurial attitude or entrepreneurial characteristics to differentiate those people who are natural entrepreneurs with many entrepreneurial characteristics, those that are not, and those who lack the characteristics mentioned [9].

In recent years, it seems clear that entrepreneurs have traits and abilities that differentiate them from other people, regardless of whether or not they undertake business activities [10]. This question leads to a new approach to the debate and a new question to consider—is the entrepreneur born or made? According to Pellicer [11], there are people who have a character that is more in line with the entrepreneurial nature, arguing that entrepreneurs frequently share some psychological traits, although these, by their very nature, are difficult to classify and generalise.

For some authors, such as Drucker [12] or Ronstadt [13], entrepreneurship is a discipline and can therefore be learned. Other authors, such as Henry et al. [14], consider entrepreneurship a science and, therefore, also think that it can be learned. In contrast, there are other authors [15,16] who believe that entrepreneurship is a talent.

Freire [17] delves deeper into this idea and states that an entrepreneur has three levels or layers: the first or most external, which are the technical habits or those characteristics that are most easily modified. At this level, you find all those variables that an entrepreneur can acquire through education; the second or intermediate layer is made up of meta-abilities or things that can be modified and learned if the entrepreneur chooses; in the third layer or level, there are the unchangeable aspects that come from the intrinsic talent that each person possesses and do not depend on external variables.

There are many approaches to the study of entrepreneurship as an attitude, but the main focus is not so much on its meaning, but on the skills that are required to develop an adequate entrepreneurial profile. From the perspective of entrepreneurship psychology, it seems clear that there are certain psychological variables and personality traits that predispose an individual to develop greater entrepreneurial skills. In addition to the most influential or predisposing personality characteristics in the development of entrepreneurial characteristics and necessary technical skills, it is important to have positive attitudes towards the development of skills. Some authors believe that the best way to teach entrepreneurial attitudes is through active learning [18], which allows the person to learn through their own experiences.

Hisrich and Peters [19] suggest entrepreneurship as a process in which entrepreneurs must acquire a series of skills that are: technical skills, such as how to communicate, or knowledge and skills in management and organization; business management skills, such as planning or making decisions; and personal skills, such as perceived internal control, innovation, risk taking, perseverance, and leadership.

Rioja et al. [20] classify 10 personal competences considered as requirements for entrepreneurial activity. These are: look for opportunities and have initiative; take risks; demand efficiency and quality; show persistence over time towards a particular goal, showing a high level of motivation; search for information; set measurable, attainable, realistic, specific, defined, and challenging goals; systematically plan and monitor the actions undertaken; be persuasive and have support networks; possess self-confidence and personal independence; and finally, commit to themselves or the projects that they carry out.

Having these skills, in addition to being something very positive and valued by organisations and the employment market today, may have other consequences. Salanova and Schaufeli [21] present results of various investigations, showing that the person’s engagement with their work activity is positively related to other variables such as extra-role behaviour [22], with personal initiative [21] and with the performance and quality of service [23]. Salanova and Schaufeli [21] point out that engagement is the psychological state that is linked to improvement and personal energy, which can affect a person´s health.

On the contrary, burnt out workers do not enjoy what they do, are stressed at work, and believe that the demands made of them are not comparable to the resources that they can count on. They face greater exposure to both physical and psychosocial risk factors for their health in the work context.

Authors such as Piqueras et al. [24] conclude that an excess of negative emotions related to the person’s own perception of competence as well as a lack of tools to combat them causes both physical and mental illnesses in the medium and long term, interfering with their normal functioning on a daily basis.

Navarro-Abal et al. [25] state that attitude and commitment towards work could influence the way in which workers perceive their health status. To test this hypothesis, these authors conducted a study among Spanish construction workers, finding correlations between job satisfaction and health perception. Some authors such as Carranza et al. [26] point out that in addition to having a positive perception of their abilities, a person must have a good perception of health, making a positive assessment of their well-being, since this will directly affect performance both physically and psychologically [27].

This data makes it possible to establish direct interdependence relationships between entrepreneurial competencies and perception of occupational health in the collective of self-employed workers.

The objective of this study is to analyse the relationship between having an entrepreneurial attitude and perceived health status among Spanish self-employed workers.

## 2. Materials and Methods

### 2.1. Design and Participants

A cross-sectional descriptive study was carried out through random sampling. A probability multistage sampling was carried out (by autonomous community, sex, age, nationality, activity sectors, working situation, and years of service).

The present study includes a cross-sectional study aimed at self-employed workers on their self-perceived health status, level of engagement, and the Personal Entrepreneurial Characteristics (PEC) during the period April 2016 and June 2017. In this period, in Spain, there were about 300,0000 self-employed or independent workers.

The sample consists of 1148 self-employed professionals from all the Spanish provinces. In relation to sex, 64.3% were men and 35.70% were women, with an age range between 18 and 72 (M = 45.29, SD = 10.06) and 42.5% had completed university studies. Also, following the National Classification of Economic Activities (CNAE-08), which distinguishes 21 economic sectors, professional, scientific and technical activities (13.91%), construction (13.48%), and wholesale and retail trade (13.20%) are highlighted in the sample. In the multiple regression analysis, the variable “economic activity” is included by grouping it in four big dimensions: primary sector, secondary sector, construction industry, and services sector. The construction industry was separated from the secondary sector in general, given its relevance within the Spanish territory. However, none of the categories included in the main variable (economic activity) have been significant in the model.

### 2.2. Assessment Tools

- Sociodemographic variables protocol. Prepared ad hoc for this research and collects information about the following variables: age, sex, level of studies, economic activity performed.
- SF36 Health Questionnaire [28]. Consists of 36 items that evaluate eight dimensions related to state of health: Physical Function, Physical Role, Body Pain, General Health, Vitality, Social Function, Emotional Role, and Mental Health.

The description of each dimension is as follows:

1. Physical Function (PF): Degree to which health limits physical activities such as self-care, walking, climbing stairs, bending, picking up or carrying weights, moderate and intense exertion.
2. Physical Role (PR): Degree in which physical health interferes with work and other daily activities, which includes lower than desired performance, limitation in the type of activities performed, or difficulty in carrying out activities.
3. Body Pain (BP): Pain intensity and its effect on routine work, both outside the home and at home.
4. General Health (GH): Personal health assessment that includes current health, future health prospects, and resistance to illness.
5. Vitality (VT): Feeling of energy and vitality versus tiredness and exhaustion.
6. Social Function (SF): Degree to which physical or emotional health problems interfere with a normal social life.
7. Emotional Role (ER): Degree to which emotional problems interfere with work or other daily activities, including a reduction of time spent on these activities, a lower than desired performance, and a decrease in attention at work.
8. Mental Health (MH): General mental health, which includes depression, anxiety, behaviour control, emotional control, and the overall positive effect.

The response options are presented in a Likert format, which assesses intensity or frequency. The number of response options varies between two, three, and six depending on the item. It shows a high internal consistency α = 0.80 for all scales, except for “social function”, which is α = 0.76.

A standard questionnaire for self-assessment of personal entrepreneurial characteristics (PEC) [20] evaluates entrepreneurial attitude. In the 1960s, in the USA, Dave Mclelland carried out studies on entrepreneurs globally to develop entrepreneurial attitudes and determine, where possible, a successful person profile. He obtained results on behaviour that characterised managers as organised, tidied persons who planned, took risks, were intelligent, and clear about what they sought, as well as creative leaders, researchers, determined people who establish good relations with others, etc. All this information was summarised in a model of 10 qualities, that is, personal entrepreneurial characteristics (PEC), which are developed in some countries, not only regarding entrepreneurs, but also students in their last semesters of their university degree, though not only related to businesses [29]. One of the most recent studies on this issue is the one by Bohm [30] from the Business School of the Andrés Bello University of Chile, which used PEC to assess entrepreneurship in the Chilean education system, or the study made by Barba [31] with managers of cooperatives from Ecuador. 

The test consists of 55 statements that are answered according to a Likert-type scale format between one (never) to five (always). The frequency with which the person identifies with each of the entrepreneurial characteristics is selected in each of the items, which in turn are distributed in three dimensions; first, the achievement dimension, which encompasses the characteristics of searching for opportunities, persistence, fulfilment of commitments, setting high standards, and taking risks; second, the planning dimension, which includes the characteristics of setting goals, obtaining information, and systematic planning; and the third, the power dimension, which includes persuasive characteristics, support networks, and self-confidence. The results of the analysis of internal consistency for this group are α = 0.76. Studies on validity and reliability have been carried out on this tool, such as the research work by Garzón [32].

### 2.3. Procedure

The sample was obtained with the collaboration of an insurance company based in Huelva, but with offices nationwide. The sample workers were “self-employed”, called “autonomous” in Spain, who represent a clearly defined figure. These workers had health insurance through an insurance company through which we had access to these workers for the study.

After several meetings with executives, authorization was obtained from the ethical commission of the company and the tests were distributed to the different Spanish offices. They were administered in the annual review carried out on a mandatory basis by the insurance company. All participants were asked for informed consent, ensuring confidentiality and anonymity of the tests. The questionnaire was completed on a personal basis in the insurance company “Activa Mutua” provincial centres.

### 2.4. Methods

A multiple regression analysis was carried out using entrepreneurial attitude as the dependent variable (derived from the PEC questionnaire), as measured both generally and according to its three dimensions: achievement, fulfilment, and power. As independent variables and following the theoretical framework, the variables are: age, sex, economic activity sector, level of studies, and the eight dimension related to self-perceived health status, i.e., physical function, physical role, body pain, general health, vitality, social function, emotional role, and mental health status.

The categorical variables (economic professional activity and level of studies) have been introduced as dummy. Value 1 represents belonging to this category and value 0 means not belonging to the category. As for the sex variable, value 1 refers to man and value 2 refers to woman.

For the regression model building, the stepwise technique was used, combining both the forward and backward techniques. First, all the variables were introduced in the model and, then, only those significant variables remained. Later, all the variables were introduced little by little if the change in R was significant.

## 3. Results

In Table 1 and Table 2, the relative frequencies and the main descriptive statistics of the variables included in the model are included.

### 3.1. Relationship between Entrepreneurial Attitude and Age, Sex, Education Level, and Professional Activity

The variables economic professional activity, level of studies, and the dimensions related to self-perceived health status: Physical Function (PF), Physical Role (PR), Body Pain (BP), Vitality (VT), and General Health (GH) have been omitted in the tables as they were not significant in the model.

As can be seen in Table 3, the results obtained show the existence of a negative linear relationship between entrepreneurial attitude and age—older participants have a less entrepreneurial attitude. With the variable sex, women display a lower entrepreneurial attitude and with mental health, the greater the entrepreneurial attitude displayed, the lower the perception of mental health. On the other hand, there is a positive linearity in social function, as people with the greatest entrepreneurial attitude score highest.

### 3.2. Relationship between the Achievement Dimension of an Entrepreneurial Attitude and Age, Sex, Educational Level, and Professional Activity

The variables economic professional activity, level of studies, and the dimensions related to self-perceived health status: Social Function (SF), Physical Function (PF), Physical Role (PR), Emotional Role (ER), Body Pain (BP), Vitality (VT), and General Health (GH) have been omitted in the tables as they were not significant in the model.

As can be seen in Table 4, there is a negative relationship between the achievement dimension of the entrepreneurial attitude and the variables age, sex, and mental health. In this way, older professionals, women, and people who perceive themselves with better mental health show lower values in the achievement dimension, i.e., in the search for opportunities, persistence, fulfilment of commitments, setting high standards, and taking risks.

### 3.3. Relationship between the Planning Dimension of an Entrepreneurial Attitude and Age, Sex, Education Level, and Professional Activity

The variables economic professional activity, level of studies, and the dimensions related to self-perceived health status: Physical Function (PF), Physical Role (PR), Body Pain (BP), Emotional Role (ER), Mental Health (MH), and General Health (GH) have been omitted in the tables as they were not significant in the model.

Table 5 indicates that there is a negative linear relationship between age and sex and the planning dimension of an entrepreneurial attitude related to setting goals, obtaining information, and systemic planning. Older professionals and women show lower values in that dimension. On the other hand, it illustrates how professionals who score higher in the planning dimension have higher values in social function and vitality.

### 3.4. Relationship between the Power Dimension of an Entrepreneurial Attitude and Age, Sex, Education Level, and Professional Activity

The variables economic professional activity, level of studies, and the dimensions related to self-perceived health status: Social Function (SF), Physical Function (PF), Physical Role (PR), Emotional Role (ER), and Body Pain (BP) have been omitted in the tables as they were not significant in the model.

Finally, Table 6 shows the negative relationship between the variables age, sex, and mental health. As a result, it is older female professionals who perceive themselves as having decreased mental health and who indicate a lower level of entrepreneurial attitude in the power dimension in terms of persuasive characteristics, support networks, and self-confidence. It also shows a positive relationship between the entrepreneurial attitude of the power dimension and vitality and general health.

## 4. Discussion

In relation to an entrepreneurial attitude and age, scientific studies point to different results, favouring diverse explanatory arguments for the results. Thus, studies such as those of Weber and Schaper [33], Kautonen et al. [34], or McKay [35] conclude that age does not constitute a barrier to entrepreneurial attitudes, emphasising that, in fact, possessing these competences provides vitality to people with an entrepreneurial attitude, although it is possible that entrepreneurs from a certain age perceive some external social discrimination towards the exteriorization of entrepreneurial behaviours. In this sense, Kautonen [36] points out that society still maintains a prejudice towards older people, seeing them as less flexible, less committed, and less able to cope with technological changes.

However, other studies, such as Katz [37], are more categorical in stating that there would be a decline in the entrepreneurial attitude of individuals as they get older. This decline, characterized by a loss of motivation to initiate or undertake new projects, actions, or activities, would begin at the end of the third decade of the individual’s life.

Other authors [38,39], however, prefer to talk about different possibilities in the relationship between entrepreneurship and age, motivated by some not so obvious results in their studies on the subject. They refer to the fact that the relationship between entrepreneurship and age is double-sided. While younger people are more likely to be entrepreneurial because they have entrepreneurial skills in the achievement dimension, which are fundamentally related to taking risks, older individuals tend towards competencies related to the power dimension, which includes the characteristics of persuasion, support networks, and self-confidence.

On the other hand, there are studies [40,41] who present the relationship between entrepreneurial attitude and age in an inverted U-shape, with the tipping point at 40 years of age.

The results of our study seem to confirm the words of Arenius and Minniti [42], when they affirm that entrepreneurship is “a young person’s game,’’ demonstrated in the analyzed sample by the fact that as age increases, the entrepreneurial attitude of the person decreases.

In relation to the sex variable, the model analysed in this work reflects results that coincide with a large number of investigations. Most of these studies have shown that there are differences in terms of sex in relation to entrepreneurial attitude, concluding that it is men who show a greater entrepreneurial attitude in different cultures and geographical areas [43]. On the other hand, Cané et al. [44] explain that these differences may be derived from the exclusion of women in the field of innovation, especially in technological contexts. They also point out the influence of the roles attributed to men, such as control and competitiveness, and women, such as cooperation and affable behaviour. In addition, in relation to individual factors, investigations such as those carried out by Fonseca et al. [45] establish that it is women who obtain the lowest score in entrepreneurial attitude associated with the greater entrepreneurial self-efficacy perceived by men, with women citing lack of knowledge, fear of failure, and doubts about performing entrepreneurial tasks.

Another variable that shows linearity in this work is vitality. Ibars et al. [46] highlight the relationship between entrepreneurship and intrinsic motivation. Thus, people with an authentic motivation have more interest and confidence and show more emotion, reflected in an elevation of vitality [47]. Along the same lines as the results found in this work, Trejo carried out an investigation in Spain using a comparative descriptive design and a sample of 229 participants. Their objective was to establish the psychosocial profile of the entrepreneur [48]. The results indicate that entrepreneurs get higher scores than non-entrepreneurs in energy, dynamism, assertiveness, determination, perseverance, stability, control of emotions, impulse control, friendliness, empathy, cordiality, conscientiousness, frankness, cultural openness, and openness to new experiences. Likewise, entrepreneurs who obtain a higher energy score have a confident and enthusiastic view of multiple aspects of life, are more dynamic, active, assertive, and dominant; they have greater emotional stability with high capacity to face the negative effects of anxiety, depression, or frustration and a greater ability to master their emotions. However, they are not very emotional, cooperative, or empathic. These last results contradict the results obtained in this research, where there is a negative linearity between entrepreneurship and mental health.

The results indicating a lower perception of mental health in those subjects with a greater entrepreneurial attitude could be explained based on their position as self-employed workers, rather than on the attitude itself. Studies such as those by Benach and Muntaner [49] suggest a relationship between self-employed work and the perceived deterioration of mental health. In a study based on cross-sectional surveys carried out in different European countries, the type of self-employment relationship was associated with a deterioration in perceived health status [50]. In fact, having a non-standardized job is related with job insecurity, which can be followed by suffering thoughts and/or suicidal actions, worse mental health, and worse self-perceived health [51]. A 2016 study by the Bellvitge University Hospital Psychiatry Service, in collaboration with the Egarsat insurance company and University of Barcelona Department of Public Health, determined that self-employment has a significant influence on mental disorders [52].

Another result found in this paper describes a positive linearity between social function and entrepreneurial attitude. It can probably be explained because people with skills directly related to commitment, motivation, and proactivity have a higher tolerance to pain and other limiting aspects related to physical and emotional health, so they do not interfere with their daily life. Pain is not only biological, as it is also composed of psychological and social characteristics and elements that, at the very least, give it a subjective significance for each person, becoming a vital individual and cross-sectional life experience [48]. Variables related to lifestyle also have an impact on the expression and subjectivity of the pain that each person suffers. According to Biedma et al. [53], behaviours and attitudes related to an active lifestyle that largely coincide with the skills that make up an entrepreneurial attitude are related to less pain and, therefore, fewer limitations which often result from pain, including social limitations.

## 5. Conclusions

The objective of the study is to analyse the entrepreneurial attitude among Spanish self-employed workers regarding their health status. The results obtained show the existence of a negative linear relationship between entrepreneurial attitude and age. The older the participants, the less likely they were to have an entrepreneurial attitude. There is also a relationship between an entrepreneurial attitude and the sex variable, with women showing the least entrepreneurial attitude. Also, regarding the relationship between an entrepreneurial attitude and mental health, the greater the entrepreneurial attitude shown, the lower the perception of mental health. In regard to the relationship of an entrepreneurial attitude with the state of health, only one positive linearity was observed with one of the dimensions of the state of health, the social function. Those with the highest entrepreneurial attitude scored higher in this dimension. Since it refers to the degree to which pain or limiting aspects of an illness affect daily social life, these results show that entrepreneurial behaviors and attitudes (related to an active lifestyle) act as a protection strategy that prevent illness from limiting the life of an entrepreneur.

The outcomes presented here are derived, in part, from the research project: “Los Determinantes Institucionales del Funcionamiento del Mercado de Trabajo en España (1939-2017). Un Estudio en Perspectiva Comparada en el Marco de la Europa del Sur” funded by the Ministry of Science, Innovation, and Universities (Reference: RTI2018-099188-A-I00).

## Figures and Tables

**Table 1:** Sociodemographic characteristics of the sample.

Characteristic	Percentage %
**Sex**	
Man	64.3
Woman	35.7
**Level of studies**	
No studies	0.9
Primary studies	19.5
Secondary studies	9.4
Higher Secondary studies	11.8
Vocational Training	15.2
University	42.5
**Economic activity**	
Primary sector	6.7
Secondary sector (excluding construction industry)	8.2
Construction industry	13.2
Services sector	71.3

**Table 2:** Descriptive statistics of the study variables.

Variable	N	Range	Minimum	Maximum	Mean	Standard Deviation
Age	1148	55	18	73	45.29	10.065
General entrepreneurial competence	1148	10.00	12.00	22.00	17.7452	1.93963
Physical function	1148	8.00	22.00	30.00	29.0810	1.75616
Physical role	1146	4.00	4.00	8.00	7.5689	1.15787
Body pain	1147	10.00	2.00	12.00	7.5058	2.26619
General health	1148	16.00	9.00	25.00	20.1592	3.25483
Vitality	1148	20.00	4.00	24.00	17.7744	4.03068
Social function	1148	9.00	2.00	11.00	10.2230	1.55316
Emotional role	1145	3.00	3.00	6.00	5.7528	0.75323
Mental health	1148	25.00	5.00	30.00	24.3859	4.31518
Entrepreneurial competence achievement	1145	11.70	10.50	22.20	17.7720	2.01776
Entrepreneurial competence fulfilment	1143	14.17	10.50	24.67	18.5992	2.35275
Entrepreneurial competence power	1125	16.00	9.00	25.00	17.4831	2.36771

**Table 3:** Model 1. Dependent variable (entrepreneurial attitude). Independent variables: age, sex, level of studies, professional activity, and health.

Age	Sex	SF	MH
−0.156 *** (0.006)	−0.093 ** (0.127)	0.093 * (0.055)	−0.124 ** (0.020)

Social Function (SF); Mental Health (MH). N = 1.148; * *p* < 0.05; ** *p* < 0.01; *** *p* < 0.001, R2: 0.048; Adjusted R2: 0.036.

**Table 4:** Model 2. Dependent variable (achievement dimension of an entrepreneurial attitude). Independent variables: age, sex, level of studies, professional activity, and health.

Age	Sex	MH
−0.144 *** (0.006)	−0.096 ** (0.132)	−0.122 ** (0.021)

Mental Health (MH). N = 1.148; ** *p* < 0.01; *** *p* > 0.001; R2: 0.048; Adjusted R2: 0.036.

**Table 5:** Model 3. Dependent variable (planning dimension of an entrepreneurial attitude). Independent variables: age, sex, level of studies, professional activity, and health.

Age	Sex	SF	VT
−0.097 ** (0.007)	−0.074 * (0.154)	0.145 *** (0.067)	0.094 * (0.025)

Social Function (SF); Vitality (VT); N = 1.148; * *p* < 0.05; ** *p* < 0.01; *** *p* > 0.001, R2: 0.048; Adjusted R2: 0.036.

**Table 6:** Model 4. Dependent variable (power dimension of an entrepreneurial attitude). Independent variables: age, sex, level of studies, professional activity, and health.

Age	Sex	VT	GH	MH
−0.142 *** (0.007)	−0.115 *** (0.156)	0.120 ** (0.026)	0.084 * (0.028)	−0.107 ** (0.025)

Body Pain (BP); Vitality (VT); General Health (GH); Mental Health (MH) N = 1.148; * *p* < 0.05; ** *p* < 0.01; *** *p* > 0.001, R2: 0.048; Adjusted R2: 0.036.
